# Anterior Hip Subluxation due to Lumbar Degenerative Kyphosis and Posterior Pelvic Tilt

**DOI:** 10.1155/2014/806157

**Published:** 2014-01-28

**Authors:** Hiroyuki Tsuchie, Shin Yamada, Hiroshi Tazawa, Hiroaki Kijima, Yoichi Shimada

**Affiliations:** ^1^Ugo Municipal Hospital, 44-5 Otomichi, Nishimonai, Ugo 012-1131, Japan; ^2^Department of Orthopedic Surgery, Akita University Graduate School of Medicine, 1-1-1 Hondo, Akita 010-8543, Japan; ^3^Japanese Red Cross Akita Hospital, 221-1 Inashirozawa, Saruta, Kamikitate, Akita 010-1495, Japan

## Abstract

Nontraumatic anterior subluxation and dislocation of the hip joint are extremely rare. A 58-year-old woman presented to our outpatient clinic with left hip pain with a duration of 15 years. There was no history of trauma or other diseases. Her hip pain usually occurred only on walking and not at rest. Physical examinations demonstrated no tenderness in the hip joint. The range of motion of both hip joints was almost normal. Laxity of other joints was not observed. The bone mineral density of the lumbar spine and proximal femur confirmed a diagnosis of osteoporosis. A plain radiograph showed osteoarthritic changes of the hip joints, severe posterior pelvic tilt, and superior displacement of both femoral heads, especially in a standing position. Three-dimensional computed tomography (3DCT) revealed anterior subluxation of both femoral heads. Seven years after the initial visit, both hip joints showed progression to severe osteoarthritis. Although the exact cause remains unclear, lumbar kyphosis, posterior pelvic tilt, and a decrease in acetabular coverage may have influenced the current case. We should be aware of these factors when we examine patients with hip osteoarthritis.

## 1. Introduction

Subluxation and dislocation of the hip joint are generally high-impact injuries, and nontraumatic anterior subluxation and dislocation are extremely rare. We describe herein a case of bilateral hip anterior subluxation possibly related to lumbar degenerative kyphosis and posterior pelvic tilt.

## 2. Case Presentation

**Figure 1:**
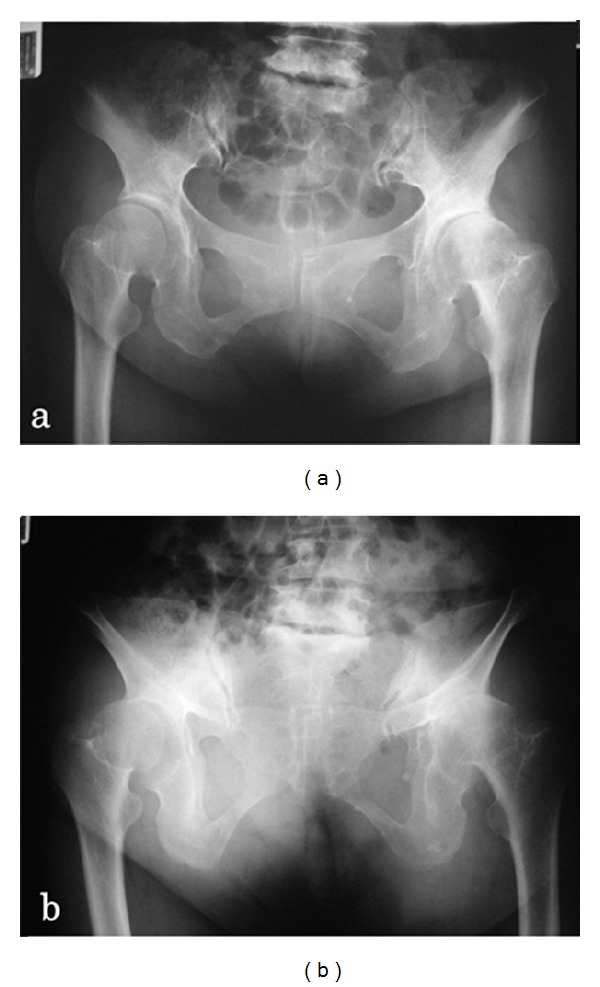
Anteroposterior radiographs of the bilateral hip joints on lying (a) and standing (b). Plain radiography showed osteoarthritic changes of hip joints and severe posterior pelvic tilt on visualizing superior displacement of both femoral heads.

**Figure 2:**
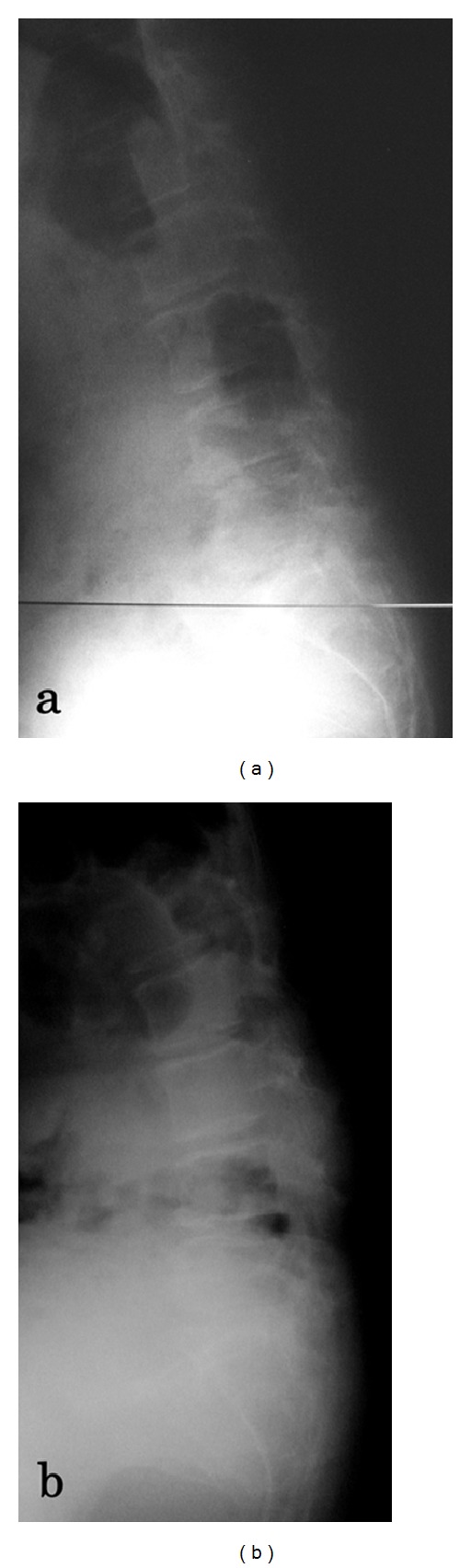
Lateral radiograph of the lumbar spine on lying (a) and standing (b). Kyphosis of the lumbar vertebrae increased on standing.

**Figure 3:**
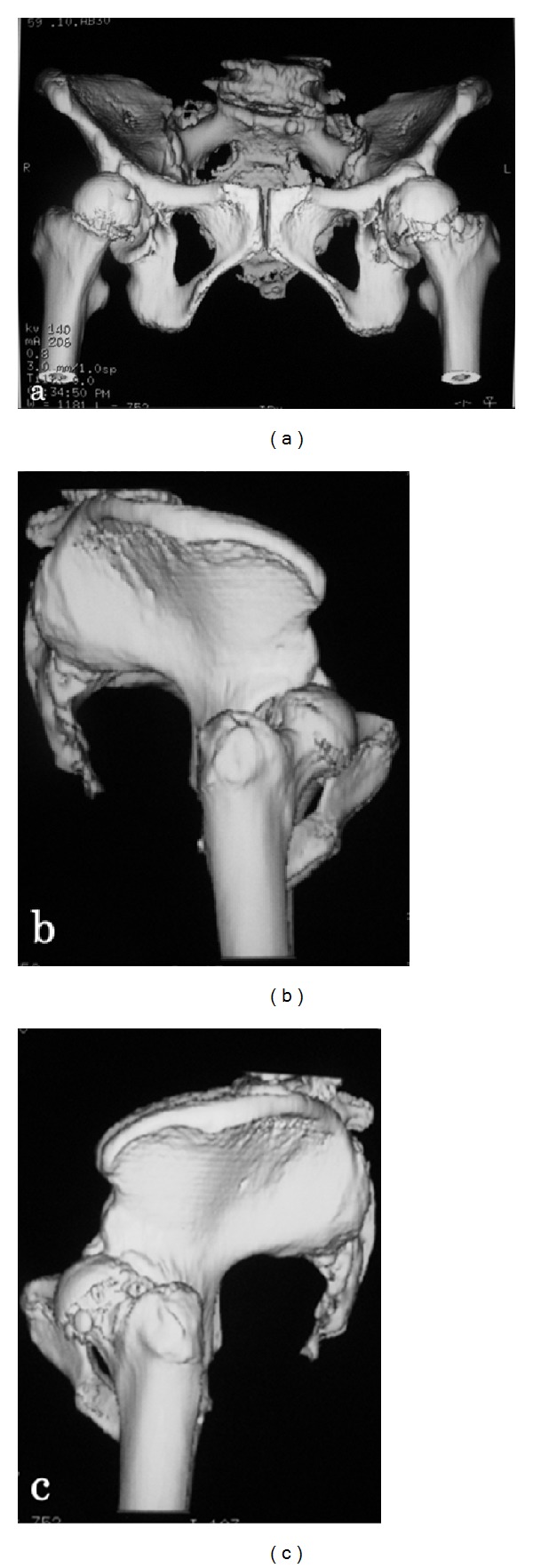
Three-dimensional computed tomography (3DCT) with an anteroposterior view (a) and lateral views of the right (b) and left (c) sides of the bilateral hip joint. 3DCT confirmed anterior subluxation of both femoral heads.

A 58-year-old woman presented to our outpatient clinic with left hip pain with a duration of 15 years. There was no history of trauma or other diseases. Her hip pain usually occurred only on walking and not at rest. Physical examination demonstrated no tenderness in the hip joint, and Patrick's fabere test was negative. The range of motion of both hip joints was almost normal: flexion was 140/140 degrees (right/left), abduction was 35/35 degrees, and adduction was 10/10 degrees. Laxity of other joints was not observed. There was no abnormal value on laboratory examinations. The bone mineral density of the lumbar spine (L2–4, 0.643 g/cm^2^, *T*-score: −3.41 S.D.) and proximal femur (0.760 g/cm^2^, *T*-score: −1.99 S.D.) confirmed a diagnosis of osteoporosis. Plain radiography of the pelvis in supine (Figure 1(a)) and standing (Figure 1(b)) positions showed osteoarthritic changes of the hip joints and severe posterior pelvic tilt on visualizing superior displacement of both femoral heads. Lumbosacral angles (LSA) on lying and standing were 27 and 6 degrees, respectively (Figure 2). Kyphosis of the lumbar vertebrae increased on standing (Figure 2). Centre-edge angles (CEA) were 17/17 degrees (right/left), Sharp angles were 36/36 degrees, and acetabular head index (AHI) values were 66/72% (Figure 1) [1]. Three-dimensional computed tomography (3DCT) confirmed anterior subluxation of both femoral heads (Figure 3). Distances from the original hip center to the migrated femoral head center were 10 mm in the right hip and 9 mm in the left hip. Nonsteroidal anti-inflammatory drugs (NSAIDs) were prescribed, and physiotherapy to train muscles around the hip joint and using a cane were recommended.

Seven years after the initial visit, both hip joints showed progression to severe osteoarthritis. We performed right total hip arthroplasty, and she could walk with a cane or walker. No problems were noted in the right hip joint, such as dislocation, at the most recent follow-up 6 years postoperatively.

## 3. Discussion

The hip joint is stable because the femoral head is widely covered with the acetabulum. So, hip dislocation and subluxation are rare. Anterior hip dislocation and subluxation are associated with neurogenic and congenital diseases [2, 3]. However, a case without such coexisting disease has not previously been reported.

**Figure 4:**
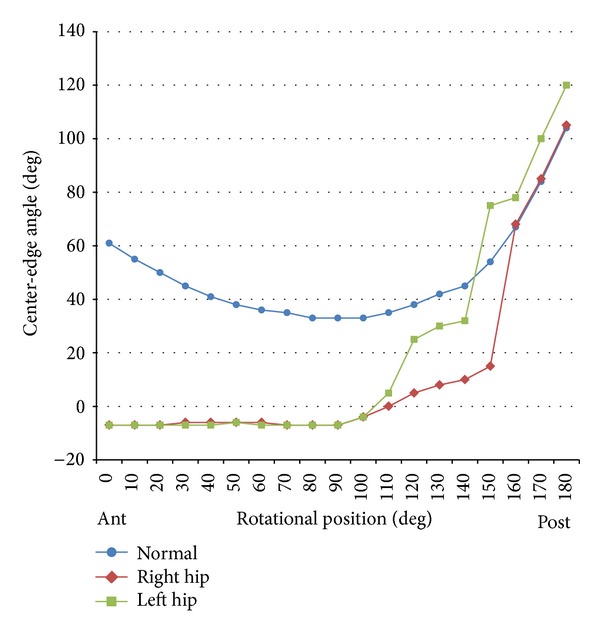
Comparative plots of the center-edge angle (CEA) measured by a transverse CT slice through the center of the femoral head for the bilateral hip joints versus mean normal values reported by Janzen et al. Our case showed very narrow acetabular coverage from the anterior to lateral area of the femoral head.

The spine, pelvis, and hip joint are closely involved with each other, and pain in the hip joint and lumbar region may be associated, referred to as hip-spine syndrome [4]. Lumbar kyphosis due to aging leads to pelvic inclination backwards to aid balance in a standing position, and pelvic posterior tilt causes a decrease in the coverage of the anterosuperior aspect of the femoral head. There are some reports on methods to evaluate anterior coverage by the acetabulum [5–9]. Janzen et al. measured CEA of a normal hip joint using vertical planar images obtained through the center point of the femoral head at various rotations from 0 (anterior acetabular margin) through 90 (lateral acetabular margin) to 180 (posterior acetabular margin) degrees by 3DCT [10]. In the present patient, 3DCT revealed a marked decrease in acetabular coverage in the anterior to lateral area of the femoral head. When we compare the acetabular coverage in the present patient with that of normal hip joints reported by Janzen et al., our patient showed very narrow acetabular coverage from the anterior to lateral area of the femoral head (Figure 4).

Acetabular dysplasia is also one of the causes of hip subluxation. The femoral head covered with a dysplastic acetabulum gradually migrates laterally or anterolaterally. In the present patient, a plain radiograph showed mild hip dysplasia (17/17 degrees) on posterior pelvic tilt. However, Sharp angles were normal (36/36 degrees) and 3DCT demonstrated no acetabular dysplasia (CE angle using the center of the acetabulum: 27/30 degrees) or other pelvic deformities in a normal position. Therefore, the most likely cause of this anterior subluxation is posterior pelvic tilt following lumbar degenerative kyphosis.

Total hip arthroplasty (THA) for patients with hip joint osteoarthritis having a posteriorly inclining pelvis occasionally leads to anterior dislocation because of increased anteversion in a standing position [11]. However, we should refrain from placing the acetabular socket to markedly reduce anteversion because there is also a risk of posterior dislocation. Rather, it is important to use a larger femoral head diameter and preserve as much soft tissue as possible.

In conclusion, this case is considered very rare, with nontraumatic subluxation anteriorly leading to osteoarthritic changes of hip joints. Although the exact cause remains unclear, lumbar kyphosis, posterior pelvic tilt, and a decrease in acetabular coverage may have influenced the present patient.
